# Inhomogeneity of the Left Atrial Strain

**DOI:** 10.3390/medicina61060944

**Published:** 2025-05-22

**Authors:** Marina Leitman, Vladimir Tyomkin

**Affiliations:** 1 Department of Cardiology, Shamir Medical Center, Zerifin 70300, Israel; 2 The Faculty of Medical & Health Sciences, Tel Aviv University, Tel Aviv 69978, Israel

**Keywords:** regional left atrial strain, left atrial displacement, regional left atrial deformation, triplane left atrial strain, left atrial function, strain, atrium, deformation, left atrium

## Abstract

*Background and Objectives*: Left atrial function is commonly assessed using speckle-tracking echocardiography, focusing on global strain averaged from the 4-chamber and 2-chamber views. However, regional variations in atrial strain remain underexplored. This study aimed to evaluate regional left atrial deformation in healthy subjects. *Materials and Methods*: A total of 22 healthy subjects underwent echocardiographic examinations during routine check-ups in 2023–2024. Images were retrieved and analyzed offline. Left atrial reservoir strain was calculated from four-chamber, two-chamber, and three-chamber views. A comprehensive map of 3-plane strain was generated for each patient, with detailed analysis of regional strain differences. Regional and average longitudinal displacement were also assessed. *Results*: There was no significant difference between triplane and biplane left atrial reservoir strain (34.4 ± 7.7% vs. 34.7 ± 6.8%, *p* = 0.9). Strain in the segments near the mitral annulus was significantly higher than in the mid-atrial segments. Mid-atrial strain was, in turn, higher than in the superior segments. Regional longitudinal displacement showed a gradient from the mitral annulus toward the superior part of the atrium, correlating well with the strain results. Additionally, strain in the inferior and septal walls was higher than in the lateral wall. *Conclusions*: Left atrial strain varies significantly across different regions. Strain is highest near the mitral annulus and lowest in the superior wall. Longitudinal displacement may serve as an additional tool for assessing left atrial function, showing a strong correlation with strain measurements. Biplane and triplane strain assessment yielded comparable results.

## 1. Introduction

The evaluation of left atrial function has traditionally centered on assessing left atrial size, primarily through echocardiography and other advanced imaging modalities, such as cardiac magnetic resonance imaging or computed tomography. These approaches have provided valuable insights into atrial dimensions but offer limited understanding of the dynamic mechanics that govern left atrial function. The left atrium plays a pivotal role in modulating left ventricular filling through a complex interplay of three distinct phases: receiving blood from the pulmonary veins, passively releasing it to the left ventricle, and actively pumping it during atrial contraction. These processes are integral to maintaining optimal cardiac output and are influenced by the intricate relationship between left atrial and left ventricular dynamics. Historically, the assessment of left atrial function relied heavily on volumetric analysis, often supplemented by Doppler-derived parameters, such as transmitral flow peak velocities and the velocity-time integral. While these metrics effectively characterize the phasic contributions of the left atrial function—the reservoir, conduit, and booster-pump phases—they lack the sensitivity to detect subtle mechanical changes or early dysfunction.

The advent of speckle-tracking echocardiography, initially developed to evaluate global and regional left ventricular function, marked a significant leap forward in cardiac imaging [1]. More recently, this technique has been adapted to assess left atrial strain, emerging as a robust and sensitive tool for quantifying the phasic mechanics of the left atrium. Left atrial strain analysis has gained recognition as an early indicator of left ventricular pathophysiology, offering a window into the atrium’s functional integrity before overt structural changes become apparent [2]. Unlike traditional metrics, strain analysis provides a detailed, deformation-based perspective on the three phases of left atrial function, each tied to specific moments in the cardiac cycle. The reservoir phase occurs during left ventricular systole, when the left atrium fills with blood from the pulmonary veins, leading to atrial expansion as the mitral annulus descends. The conduit phase follows in early diastole, characterized by passive left ventricular filling as blood flows from the left atrium to the left ventricle along a pressure gradient, with the atrium shortening in the process. Finally, the booster-pump phase involves active atrial contraction in late diastole, further augmenting left ventricular filling and reflecting the left atrial contractile capacity [2]. These phases collectively highlight the left atrium’s dynamic role in supporting left ventricle performance.

Left atrial phasic function, particularly reservoir strain, has emerged as a sensitive and early marker of atrial dysfunction, often preceding changes in size or volume. The strain traces derived from speckle-tracking imaging reveal a close mechanical interdependence between the left atrium and left ventricle, largely due to their shared connection at the mitral annulus [3]. This reciprocal relationship is evident throughout the cardiac cycle: as the left atrium fills during the reservoir phase, the left ventricle empties, and as the left atrium empties during the conduit and booster phases, the left ventricle fills. Consequently, left atrial strain measurements are intricately linked to left ventricular mechanics, with studies demonstrating that alterations in left ventricular compliance or pressure directly influence left atrial strain values [3,4]. Notably, left atrial reservoir strain has been shown to correlate strongly with left ventricular filling pressures, serving as a non-invasive surrogate for hemodynamic parameters. Smaller studies have further substantiated its clinical relevance, identifying associations between peak left atrial strain and invasive measures such as pulmonary capillary wedge pressure and left ventricular end-diastolic pressure, suggesting its potential utility in diagnosing conditions like heart failure with preserved ejection fraction or atrial fibrillation [5,6,7].

Left atrial strain in patients with atrial fibrillation has been shown to correlate with stroke risk [8,9]. In patients with stroke, reduced left atrial strain has also been associated with the subsequent detection of atrial fibrillation, being particularly valuable in those with a normal left atrial size [9]. In this context, a detailed evaluation of regional left atrial function may serve as an early and sensitive marker of left atriopathy. As a marker of atrial fibrosis, left atrial strain has also been identified as a predictor of clinical outcomes and functional capacity in patients with severe primary mitral regurgitation [10]. Moreover, low left atrial reservoir strain has been found to correlate with atrial fibrosis-related COL1A1 gene expression in patients undergoing cardiovascular surgery [11].

Despite the growing appreciation for left atrial strain as a diagnostic tool, regional variations in strain remain underexplored in the literature. Most studies to date have focused on global left atrial strain or employed biplane methods to assess regional differences [12,13], while others have utilized three-dimensional echocardiography to estimate average wall strain [14]. However, these approaches often oversimplify the left atrium’s complex, heterogeneous structure, potentially missing localized abnormalities that could signal early pathology. Detailed investigations into triplane left atrial strain—leveraging multiple imaging planes to capture regional mechanics—are notably scarce, limiting our understanding of how strain varies across different segments of the atrium. This gap in knowledge underscores the need for more refined methodologies to fully elucidate left atrial function.

In this study, we aimed to address these limitations by assessing regional left atrial strain using a triplane method in a cohort of healthy young individuals. This approach allows for a more comprehensive evaluation of left atrial mechanics by integrating data from three distinct echocardiographic planes, offering greater spatial resolution than traditional biplane or global analyses. Furthermore, we explored longitudinal peak displacement during the reservoir phase as a novel deformation parameter, a metric that has not been previously reported in the literature for evaluating left atrial function. By examining both strain and displacement in a healthy population, this study sought to establish normative values and provide a foundation for future investigations into pathological left atrial remodeling, potentially enhancing the early detection of cardiovascular disease.

## 2. Materials and Methods

### 2.1. Study Population

A total of 22 young, physically active healthy individuals underwent routine check-ups in 2023–2024 at our hospital. Echocardiographic examinations were retrieved and analyzed offline. Left atrial reservoir strain was calculated from 4-chamber, 2-chamber, and 3-chamber views. A visual plot of the 3-plane strain was generated for each patient and analyzed in detail for regional strain differences. Longitudinal displacement, both average and regional, was also obtained and analyzed similarly.

### 2.2. Echocardiographic Assessment

The echocardiographic assessment was conducted according to current guidelines. All studies were performed using the VIVID E95 v203 (GE Healthcare, Horten, Norway) echocardiography system, with digital storage of all examinations. Standard assessments included linear and volumetric measurements of the cardiac chambers and evaluation of diastolic function. Diastolic function parameters included mitral inflow velocities (E and A waves), E-wave deceleration time, E/A ratio, and tissue Doppler imaging of the mitral annular septal and lateral velocities. Additionally, LA strain and displacement were calculated. Left atrial strain was evaluated according to the recent consensus document published by the European Association of Cardiovascular Imaging [3], using 2D speckle-tracking echocardiography. Optimal, non-foreshortened apical views were used, with the reference time point set at left ventricular end-diastole (strain = 0), determined by the mitral valve inflow. The atrial wall endocardial border was delineated. Reservoir strain was defined as the peak strain before the opening of the mitral valve during LV systole (LASr) [3]. Both automatic biplane and manual triplane methods were utilized.

#### 2.2.1. Automatic Biplane Method for Left Atrial Strain [15]

The 4-chamber and 2-chamber apical views were used by the automated software. Average peak reservoir strain was calculated and reported.

#### 2.2.2. Manual Triplane Left Atrial Strain Assessment Using Ventricular-Dedicated 2D Strain Software

LA longitudinal strain was manually calculated from 4-chamber, 2-chamber, and 3-chamber views. Average and regional peak reservoir strain values were reported.

#### 2.2.3. Left Atrial Walls and Segment Definitions for Regional Strain Calculation

**Figure 1:**
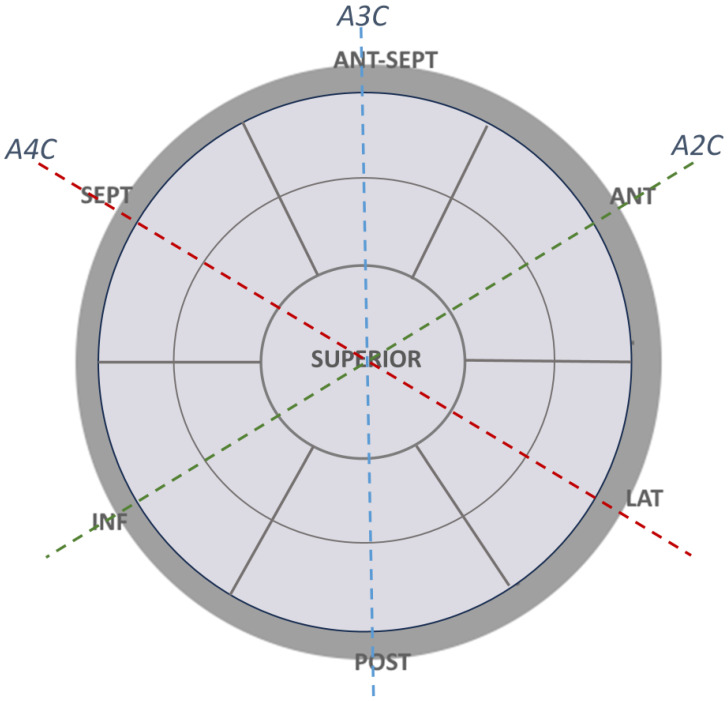
Schematic presentation of the left atrium. Seven walls were defined: septal, antero-septal, anterior, lateral, posterior, inferior, and superior. Outer ring—basal segments, middle ring—mid-segments, inner circle—superior segments. Red, green and blue punctuated lines correspond to 4-chamber, 2-chamber, and 3-chamber projection of the left atrium, respectively. A4C—apical 4-chamber view, A2C—apical 2-chamber view, A3C—apical 3-chamber view.

For this study, 7 walls were defined: septal, antero-septal, anterior, lateral, posterior, inferior, and superior (Figure 1). Segments near the mitral annulus were classified as basal segments, while those at mid-level were classified as mid-atrial segments. The average peak reservoir strain of the superior segments was calculated and reported [16], considering the anatomy of the superior left atrial wall [16]. Average triplane strain was compared with biplane strain, and regional reservoir strain was calculated and reported for each segment.

#### 2.2.4. Longitudinal Displacement

**Figure 2:**
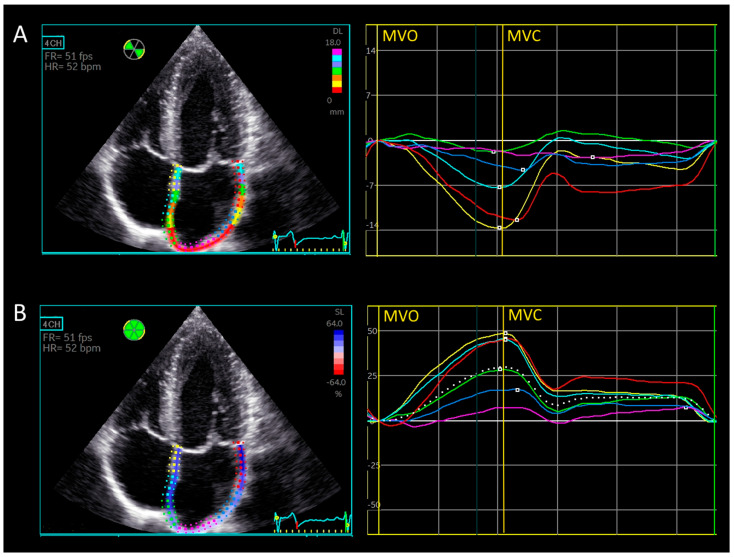
Longitudinal displacement and strain of the left atrium. (**A**) Apical 4-chamber view, longitudinal displacement curves from six segments are shown. MVO—mitral valve opening. MVC—mitral valve closure. (**B**) Apical 4-chamber view. Regional and global longitudinal strain from left atrium is shown. MVO—mitral valve opening. MVC—mitral valve closure.

Longitudinal displacement refers to the simple absolute displacement of a segment during the cardiac cycle, measured in millimeters. After calculating triplane strain, longitudinal displacement was obtained using the same software and calculated as an average over the entire atrium. Average and regional longitudinal displacement during the reservoir phase (LADr) was reported. Figure 2A displays the regional longitudinal displacement of left atrial segments from the 4-chamber view, with regional and global left atrial strain curves from the same view shown in Figure 2B for better visualization.

### 2.3. Statistical Methods

Descriptive statistics were computed to summarize each parameter’s characteristics. Continuous data are presented as means ± standard deviations. The normal distribution of differences was assessed using the Kolmogorov–Smirnov test. A two-tailed, dependent t-test was used for the continuous variables. Categorical data are reported as numbers and percentages. Univariate analysis was conducted using the Chi-Square test or Fisher’s exact test, as appropriate, to determine the significant variables (*p* < 0.05). Statistical analysis was performed using IBM SPSS Statistics for Windows, Version 28.0 (Armonk, NY, USA: IBM Corp).

### 2.4. Ethical Approval

Formal approval by the Ethics (Helsinki) Committee at Shamir (Assaf Harofeh) Medical Center was not required for this retrospective study. Informed consent was also not applicable due to the anonymized nature of the data. All echocardiographic images included in the manuscript were fully de-identified to ensure patient confidentiality. All methods were conducted following relevant guidelines and regulations.

## 3. Results

### 3.1. Age, Anthropometric and Echocardiographic Data

The age, anthropometric, and echocardiographic characteristics of the study participants are summarized in Table 1. The mean age of the participants was 24 ± 4.3 years. The mean left ventricular ejection fraction was 58.7 ± 2.6%, with no regional wall motion abnormalities observed. The mean left atrial volume index was 33.3 ± 9.3 mL/m^2^, and all diastolic function parameters were within normal limits.

### 3.2. Strain Analysis

**Figure 3:**
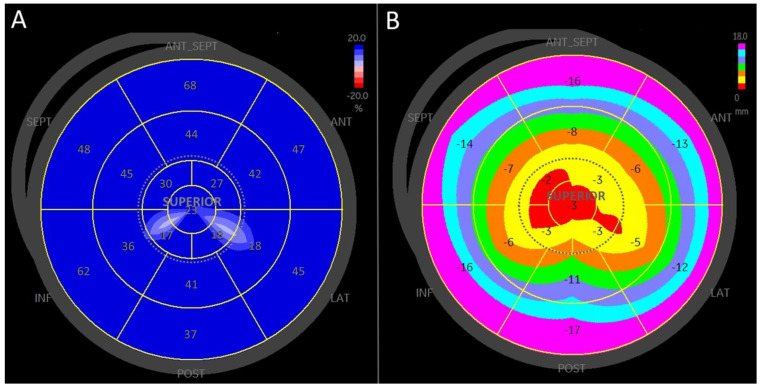
A bullseye plot of left atrial longitudinal strain and displacement in a healthy 23-year-old male demonstrates a gradient from basal to superior segments. ANT-SEPT—antero-septal wall, SEPT—septal wall, INF—inferior wall, POST—posterior wall, LAT—lateral wall, ANT—anterior wall, SUPERIOR—superior wall. (**A**) Left atrial regional longitudinal reservoir strain map. The map shows higher strain values in the basal segments compared to the mid-atrium, with the mid-atrium exhibiting higher strain than the superior wall. (**B**) Left atrial regional longitudinal reservoir displacement map. Longitudinal displacement is greater in the basal segments compared to the mid-atrium, with displacement in the mid-atrium being higher than in the superior segments.

Strain analysis data are presented in Table 2. The average biplane reservoir strain was 34.4 ± 7.7%, which correlated well with the triplane reservoir strain of 34.7 ± 6.8% (*p* = 0.9). Regional strain differences from the basal to superior segments are detailed in Table 2 and illustrated in Figure 3.

The highest regional peak reservoir strain was observed in the basal (annular) segments at 46.1 ± 16.9%, which was significantly higher than the peak reservoir strain from the mid-atrial segments (37.7 ± 13.7%; *p* < 10^−5^). Additionally, peak reservoir strain from the mid-atrial segments was significantly higher than the average strain from the superior wall (27.5 ± 7.6%; *p* < 10^−7^).

### 3.3. Longitudinal Displacement

Longitudinal displacement data are presented in Table 2 and Figure 3. Peak reservoir longitudinal displacement exhibited a gradient from the basal to the superior segments, with maximal displacement at the basal (annular) segments and the lowest displacement at the superior segments.

### 3.4. Left Atrial Wall Motion Analysis

**Figure 4:**
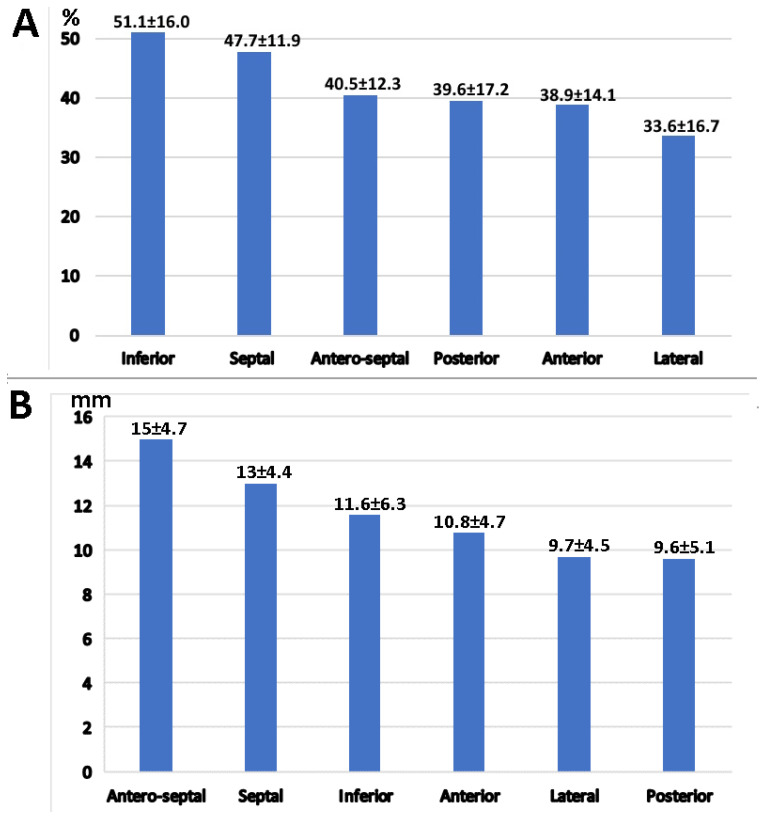
Regional reservoir strain and longitudinal displacement across the left atrium. (**A**) Regional reservoir strain (%) across the left atrium. Inferior and septal segments exhibit the highest strain values. Strain in the inferior and septal segments was significantly higher than in the other segments (*p* < 0.01). (**B**) Regional reservoir displacement (mm) across the left atrium. Antero-septal displacement was significantly higher than in the other segments (*p* = 0.04).

The inferior wall exhibited the highest strain at 51.1 ± 16.0%, followed by the septal wall strain at 47.7 ± 11.9% (Figure 4). The greatest strain values were observed in the inferior and antero-septal segments (Figure 4). Similarly, the highest longitudinal displacement was noted in the antero-septal, septal, and inferior segments (Figure 4).

**Figure 5:**
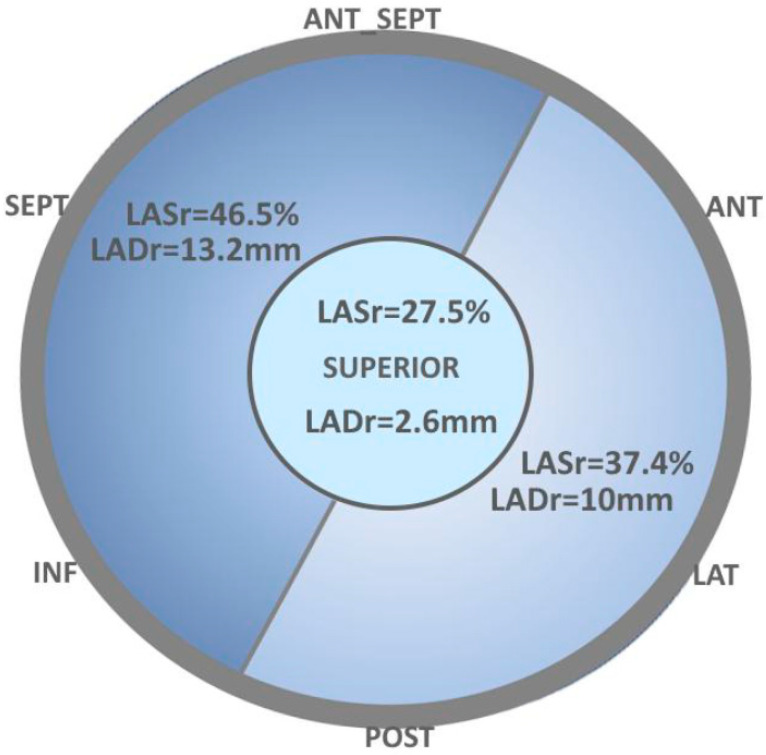
Comparison of average left atrial reservoir strain and displacement between the antero-septal, septal, and inferior segments versus the anterior, lateral, and posterior segments. Average reservoir strain and displacement were significantly higher in the septal and inferior segments compared to the anterior, lateral, and posterior segments (*p* < 0.00001). The reduction in strain and displacement from the basal to the superior part of the left atrium is depicted with a color gradient.

Both the reservoir strain and longitudinal displacement across the septal, antero-septal, and inferior segments were significantly higher compared to the anterior, lateral, and posterior segments (46.5 ± 14.2% vs. 37.4 ± 16.3%; *p* < 10^−5^, and −13.2 ± 5.4 mm vs. −10 ± 4.8 mm; *p* < 10^−5^), as shown in Figure 5.

## 4. Discussion

In this study, we aimed to evaluate regional variations in left atrial strain and longitudinal displacement using a triplane approach in a cohort of 22 healthy subjects. Our findings reveal significant inhomogeneity in left atrial strain across different segments, with a clear gradient from the basal segments near the mitral annulus to the superior segments. This pattern was consistently reflected in longitudinal displacement, underscoring the utility of both parameters in assessing left atrial function.

Average left atrial reservoir strain has been extensively investigated [17,18,19,20,21], with evaluations conducted across various clinical settings, including heart failure [20,22], diastolic function assessment [6,23,24], aortic stenosis [25], and mitral regurgitation [26], and as a predictor of atrial fibrillation [21,27].

While average strain assessment is commonly used in research and sometimes in clinical practice, typically utilizing one or two echocardiographic views [3], our study is the first to apply a detailed triplane method for regional strain analysis across the entire left atrium. The inclusion of the three-chamber view provides a more comprehensive assessment, offering improved visualization of segments like the superior wall, which is often underrepresented in biplane methods.

Regional atrial strain has been minimally investigated. One previous study [12] indicated that left atrial strain tends to be higher in the basal segments closest to the mitral annulus. This study focused on biplane strain using only two echocardiographic views. In contrast, our study provides a comprehensive analysis of regional strain across the entire atrium and reveals a gradual decrease in strain and longitudinal displacement from the basal segments to the superior wall. The physiological basis for this observation is the close functional relationship between the left atrium and left ventricle. During the reservoir phase, the basal segments experience greater mechanical forces due to their proximity to the mitral annulus, resulting in higher strain values. Conversely, the superior segments, which are less directly involved in left ventricular filling dynamics, exhibit lower strain.

The regional differences observed in left atrial strain may have important implications for clinical practice. Conventional echocardiographic assessments that rely on average biplane strain may overlook significant regional variations, which can be clinically relevant, especially in conditions such as atrial remodeling, fibrosis, or pressure overload. Recognizing these variations is crucial for more accurate interpretation of strain measurements, particularly in patients with atrial fibrillation, diastolic dysfunction, or heart failure.

There is growing evidence that atrial cardiomyopathy represents a complex of structural, functional, anatomical, and electrophysiological changes affecting the atria, which may have clinically important manifestations. The development of atrial cardiomyopathy is mediated by genetic, inflammatory, metabolic, and environmental factors, and is influenced by aging and gender differences.

Catheter ablation of atrial fibrillation is the most reliable rhythm control strategy and may even result in partial reversal of atrial fibrillation-induced atrial cardiomyopathy [28]. Catheter ablation has become a standard of care for atrial fibrillation in appropriate patients and is performed based on electro-anatomical mapping. A correlation has been demonstrated between the extent of low-voltage areas, as estimated by ultra-high-density mapping, and left atrial strain, with both parameters serving as indicators of atrial fibrosis and atriopathy [29]. Regional left atrial function assessed by strain and displacement may be valuable in patients undergoing catheter ablation, providing a non-invasive preprocedural assessment tool and allowing prediction of ablation success in atrial fibrillation patients.

Other imaging modalities, such as cardiac magnetic resonance, can assist in the assessment of left atrial size and fibrosis. Recently, a retrospective gated computed tomography algorithm has been proposed to generate three-dimensional imaging of cardiac anatomy throughout the cardiac cycle, allowing the calculation of global and regional reservoir strain [30].

Additionally, our study demonstrates a strong correlation between longitudinal displacement and strain, suggesting that this easily measurable parameter could serve as an adjunct in evaluating left atrial function. Longitudinal displacement is straightforward to obtain and may be particularly useful in cases where strain analysis is challenging due to poor image quality or suboptimal tracking.

Longitudinal displacement could serve as a useful complementary parameter in cases where strain tracking is limited due to suboptimal image quality or technical limitations of speckle-tracking. Since displacement measurements rely primarily on tracking of anatomic landmarks such as the atrioventricular annulus, they may be more robust in challenging imaging conditions. Additionally, assessing displacement may provide further insights into regional atrial mechanics and contribute to a more comprehensive evaluation of left atrial function.

This study boasts several notable strengths, including the adoption of a triplane approach, which facilitates a more thorough and comprehensive assessment of strain compared to traditional methods. Additionally, the study benefits from its focus on a homogeneous population of young, healthy individuals, effectively minimizing the influence of confounding variables such as age-related comorbidities or lifestyle factors that could skew the results. Conventional monoplane left atrial measurements, such as atrial diameter, often fall short in providing an accurate representation of left atrial function due to their limited scope and inability to capture the atrium’s complex geometry. Previous research has highlighted these shortcomings, proposing the use of an eccentricity index derived from multiplane left atrial diameter measurements as a more reliable method for evaluating left atrial size, function, and remodeling [31,32]. Building on this foundation, the triplane approach to the left atrial strain employed in this study offers a significant advancement by enabling a multi-dimensional analysis of left atrial strain. This method serves as a valuable complement to existing techniques, enhancing the precision of left atrial function assessment and offering potential insights into the early detection and evaluation of pathological left atrial remodeling, which could have implications for conditions such as atrial fibrillation or heart failure.

However, some limitations must be acknowledged. The relatively small sample size may limit the generalizability of our findings. Additionally, since this study focused on healthy male subjects, the results may not directly apply to clinical populations with atrial pathology.

Future research should explore regional left atrial strain in larger and more diverse populations, including those with known cardiovascular conditions, such as heart failure or atrial fibrillation, and including both genders to better understand potential sex-based differences in left atrial strain and displacement.

Additionally, the use of software originally developed for ventricular strain assessment to analyze left atrial strain represents a methodological limitation, but prior to the development of automated algorithms specifically for left atrial strain, it was common practice in the field to apply ventricular software to left atrial strain measurements, particularly in two- and two-chamber views. Although triplane analysis is less validated, the application of the same approach to three-chamber views appears reasonable.

Manual nature of the triplane analysis introduces potential variability. We acknowledge this as a limitation. However, previous studies using similar manual speckle-tracking techniques have demonstrated acceptable levels of reproducibility, with inter- and intra- observer variability of less than 5.5% [33,34,35].

Further research is needed to investigate the role of regional left atrial strain patterns in patients with atrial diseases. Future studies should assess whether specific strain patterns correlate with outcomes in conditions such as atrial fibrillation or heart failure with preserved ejection fraction. Additionally, longitudinal studies involving athletes could provide insights into how left atrial strain patterns evolve over time and whether they predict the development of atrial pathology later in life.

## 5. Conclusions

In conclusion, our study highlights the inhomogeneity of left atrial strain across different segments of the left atrium, with the highest strain observed in the basal segments and the lowest in the superior segments. Longitudinal displacement also correlates well with strain and could serve as a useful parameter for assessing left atrial function. These findings underscore the importance of considering regional variations in strain when interpreting echocardiographic data, which may have significant clinical and research implications.

## Figures and Tables

**Table 1:** Age, Anthropometric, and Echocardiographic Data.

Variable	Value
Age, years	24 ± 4.3
Height, cm	179.7 ± 8.8
Weight, kg	74.2 ± 6.2
BSA m^2^	1.93 ± 0.13
LAVi, mL/m^2^	33.3 ± 9.3
LVEDD, cm	5.03 ± 0.37
LVESD, cm	3.04 ± 0.38
IVS, cm	0.95 ± 0.09
PW, cm	0.95 ± 0.1
LVMi, g/m^2^	84.5 ± 15.9
EF, %	58.7 ± 2.6
E/A ratio	1.9 ± 0.6
Edec, msec	161.8 ± 40.1
E/E’ ratio	5.03 ± 0.89

BSA—body surface area, LAVi—left atrial volume index, LVEDD—left ventricular end-diastolic diameter, LVESD—left ventricular end-systolic diameter, IVS—interventricular septum, PW—posterior wall, LVMi—left ventricular mass index, EF—ejection fraction, E/A—E to A ratio, E deceleration—deceleration time of E.

**Table 2:** Regional reservoir left atrial strain and displacement.

Segment	Strain, %	*p*-Value	Displacement, mm	*p*-Value
Basal	46.1 ± 16.9	NA	15.2 ± 4.0	NA
Mid-atrium	37.7 ± 13.7	1.6 × 10^−5^	8 ± 4.0	<10^−35^ †
Superior	27.5 ± 7.6	<10^−7^ ‡	2.6 ± 1.0	<10^−9^ ‡

Reservoir strain in basal segments is higher than in the mid-atrium. Reservoir strain in the mid-atrial segments is higher than in the superior segments. Reservoir displacement in basal segments is higher than in the mid-atrium, and reservoir displacement in the mid-atrium is higher than in the superior segments. Both the reservoir strain and displacement exhibit a basal-to-superior gradient, with *p*-value even higher with displacement; †—*p*-value represents the difference between the mid-atrium and basal segments; ‡—*p*-value represents the difference between the superior and mid-atrium segments.

## Data Availability

The data supporting the findings of this study are available from the corresponding author upon reasonable request.
